# Crack reduction in laser powder bed fusion of MnAl(C) using graphene oxide coated powders

**DOI:** 10.1038/s41598-024-51283-5

**Published:** 2024-01-11

**Authors:** Simon Tidén, Himesha Abenayake, Julia Löfstrand, Ulf Jansson, Martin Sahlberg

**Affiliations:** 1 Department of Chemistry-Ångström Laboratory, Uppsala University, Box 538, 751 21 Uppsala, Sweden; 2 Department of Physics and Astronomy-Ångström Laboratory, Uppsala University, Box 516, 751 20 Uppsala, Sweden

**Keywords:** Metals and alloys, Magnetic materials, Design, synthesis and processing

## Abstract

MnAl(C) is a promising candidate as a rare earth free magnet. When processing MnAl(C) in laser powder bed fusion (L-PBF) the high cooling rates can retain the high temperature ε-phase which can then be annealed at low temperatures to yield the ferromagnetic τ-phase. However, MnAl(C) has been shown to be difficult to print using L-PBF and the material is prone to severe cracking. In this study, we have investigated how the addition of a graphene oxide (GO) coating on the powders can affect the processability of MnAl(C) and properties of the printed parts. MnAl(C) powders were coated with 0.2 wt.% GO using a wet-chemical process. The addition of GO reduced crack formation in the printed parts, and also influenced the degree of $\langle {001} \rangle$ texture along the build direction. After printing, densities of 93% and 87% could be achieved for the reference and 0.2 wt.% GO, respectively. Furthermore, a 35% reduction of cracking was calculated from image analysis, comparing printed samples produced from coated and non-coated powders. Both powders formed mostly the ε-phase but some two-phase regions with a mix of γ- and ε-phase could be observed in the as-printed parts, but seemed to be more prominent in the uncoated reference samples and could also be linked to cracks. The τ-phase together with smaller amounts of secondary phases was obtained after heat treatment at 560 °C for 5 min for both samples. Vibrating sample magnetometry was used to measure the magnetic properties, the reference had a remanence of 33 Am^2^/kg and a coercivity of 139 kA/m, and the 0.2 wt.% GO sample showed a similar remanence of 30 Am^2^/kg and coercivity of 130 kA/m. These results show that GO coating is a viable method to reduce detrimental cracking in L-PBF MnAl without reducing the magnetic performance of the material.

## Introduction

Magnets are a vital part of the green energy transition and are used in for example electric motors and wind turbines. However, all rare earth (RE) elements are not sustainable from a supply chain perspective, and they are also expensive due to the complex refining process^1^. Since the RE material crisis in 2012, prices have soared over 900% and spurred a search for cheaper, and more sustainable alternatives^2^. Among the RE-free magnets, MnAl is a potential materials system with promising properties^3^. Each Mn atom has five 3D electrons that can result in large magnetic moment. But elemental Mn exhibits antiferromagnetic ordering due to nearly half-filled 3D orbitals and direct exchange coupling between Mn–Mn atoms when the distance between the Mn atoms is too small^4,5^. However, when alloying with Al to form the MnAl compound, ferromagnetic coupling can be obtained, needed for permanent magnets^6^. This can be found in the metastable τ-phase, which requires a Mn content between 66.5–75.0 wt.%^7,8^. The tetragonal τ-phase can be obtained by either rapid cooling from the hexagonal ε-phase followed by annealing at ~ 450–650 °C or controlled cooling directly from the ε-phase^9,10^. The metastable τ-phase can with additional annealing decompose into the thermodynamically stable, but non-ferromagnetic, β-phase and γ_2_-phase. Consequently, to optimize the magnetic properties, MnAl magnets should consist of the τ-phase with minimal amounts of either ε-phase, or the stable β- and γ_2_-phases.

The stability of the τ-phase^7,11–13^ can be improved by the addition of carbon but this will not fully prevent the formation of β-phase and γ_2_-phase^10^. The solubility limit of carbon in the τ-phase is 0.9 wt.% and an increase in τ-phase stability was observed up to the solubility limit. Furthermore, carbon addition can also reduce stresses in the material but leads also to a reduced Curie temperature (T_c_)^14^. Other factors influencing the τ-phase formation and stability is the annealing temperature of the ε-phase and the microstructure^15–17^. Higher annealing temperatures and a finer microstructure facilitates the ε–τ transformation as well as the decomposition into the thermodynamically stable β-phase and γ_2_-phase. However, it is not only the τ-phase purity that determines the magnetic properties of the material. Other factors such as microstructure and defect density also influence the magnetic properties. For example, plastically deformed samples with high dislocation density could show increased coercivities^18^ and magnetic anisotropy can be introduced by ball milling powders into flakes^19^.

Additive manufacturing presents a great opportunity in the field of permanent magnets as it is envisioned to enable the manufacture of complexly shaped magnets with enhanced properties, and also the possibility of increasing the anisotropy through texture, which can enhance the overall magnetic coercivity. Radulov et al.^20^ used electron beam melting (EBM) to produce fully dense (5.1 g/cm^3^) MnAl-based magnets. The as-printed composed of mainly the γ_2_-phase but a two-step annealing program at 1100 °C for 60 h followed by 500 °C for 30 min achieved 90% τ-phase with magnetization up to 100 Am^2^/kg at 2 T applied field, BH_max_ of 1.85 kJ/m^3^ and coercivities around 80 kA/m. Krakhmaley et al.^21^ did line scans using laser powder bed fusion (L-PBF) on an Al build plate using laser powders between 50–350 W, scanning speeds 80–2200 mm/s. The lowest and highest laser power yielded irregular or delaminated melt tracks, all samples were prone to cracking which was reduced with higher laser powers. The main as-printed phase was the ε-phase, with higher laser powers showing more secondary phases (γ_2_, β and small amounts of τ), presumably because of larger heat affected zone which would allow more time for the phases that are stable at lower temperatures to form.

Pacheco et al.^22^ used L-PBF to print $\langle {001} \rangle$ textured MnAl(C) samples along the build direction with high purity ε-phase which was heat-treated at 580 °C for 5 min followed by water quenching. This yielded the τ-phase but with non-magnetic impurity phases (β, Mn_3_Al(C), γ_2_) which led to saturation magnetization of only 39.3 Am^2^/kg, a coercivity of 168 kA/m and remanence of 17.5 Am^2^/kg. A low laser power (18–25 W) re-melting strategy combined with slow scanning speeds between 160–240 mm/s was employed to improve processability, however, the highest density was reported as 4.4 g/cm^3^ (~ 88%). Such a low density will bring down the volume magnetization significantly. They also observed a severe cracking of the printed samples, which limits the use of this material in practical applications.

Previous trials with EBM and L-PBF of MnAl(C) clearly show that cracking has to be reduced. In this study, we will show that powder coated with graphene oxide (GO) will give a lower crack formation in L-PBF of MnAl(C). Graphene oxide (GO) in small amounts (0.1–0.3 wt.%) has been used to print other difficult-to-process materials such as Cu due to an improved absorption from the laser^23^. Graphene oxide can also be a carbon source making it possible to use pure MnAl powder. The aim of this study is to investigate how a GO coating on the MnAl(C) powders can affect the L-PBF printing process and the crack behavior, and additionally investigate how the GO addition affects phases and texture formed in the as-printed parts as well as the subsequent τ-phase transition and magnetic properties.

## Experimental

Gas-atomized MnAl(C) powder (Höganäs AB) was used in this study. The powder had the composition 72.2 wt.% Mn, 27.2 wt.% Al, 0.61 wt.% C and 0.012 wt.% O and the particle size was below 63 µm. The size distribution of the powders is shown in supplementary information (Table SI 1 and Fig. SI 1). The powder was coated with 0.2 wt.% graphene oxide (GO) by Graphmatech AB using a wet-chemical process using pH-dependent electrostatic forces to coat the surface of the MnAl(C) powder particles. The coating procedure has been described in more detail in a previous publication^24^. A pH of 7.4 was chosen to coat GO on MnAl(C), based on Al_2_O_3_ being the dominating surface oxide^25^, it is stable between pH ~ 3.9 and ~ 8.6^26^ and has a positive surface charge below pH 8.5–9.5^27,28^ which can attract the negatively charged GO^24^.

The printing was performed using an EOS 100M L-PBF printer equipped with a 200 W fiber laser to print 8 mm diameter and 6 mm high cylinders (Fig. SI 2). The chosen process parameter range was based on previous work to print MnAl without GO coating^22^. Layer thickness and hatch distance were kept at 20 µm and 70 µm, respectively. The laser power was varied between 20–100 W and the scanning speeds between 200–500 mm/s, including re-melting each layer was investigated, but only prints using 20–22 W and 200–300 mm/s could be completed. All prints were done on a 316L stainless steel build plate, a re-coater blade and without substrate heating. The scanning pattern was a 5 mm striped raster pattern rotating 67° every layer while avoiding the scans with laser paths parallel to the protective Ar gas flow. The oxygen content in the build chamber was below 0.12% of the total gas pressure for all prints. All characterization besides density measurements were done on samples printed with 20 W laser power and 240 mm/s scanning speed.

Phase composition of powders and printed parts were analyzed with X-ray diffraction (XRD) using a D8 Bruker Powder diffractometer using Cu-Kα radiation operated at 40 kV and 40 mA. XRD on the polished cross-sections of the printed parts was done with the diffraction vector parallel to the build direction. Rietveld refinement was done using the TOPAS software (https://www.bruker.com/en/products-and-solutions/diffractometers-and-x-ray-microscopes/x-ray-diffractometers/diffrac-suite-software/diffrac-topas.html) version 6^29^. MnAl phase diagram was calculated using Thermo-calc software (version 2019b) and the TCFE9 v9.1 database^30^. Grinding was done using SiC paper followed by polishing using 9 µm, 3 µm, 1 µm diamond particle suspensions and a final step of colloidal silica polishing. Etching was done by swabbing with Keller’s reagent (H_2_O:HNO_3_:HCl:HF in ratios 95:2.5:1.5:1). Characterization of the powder, GO coating and microstructure of the printed parts were done using a Zeiss LEO 1530 scanning electron microscope (SEM) with a Schottky field emission gun (FEG) equipped with an 80 mm^2^ silicon drift energy dispersive X-ray spectroscopy (EDS) detector. Density, area of cracks, cell width of printed parts and particle-size distribution of powders was calculated using ImageJ analysis of SEM micrographs. The width of the cells in the cellular microstructure was estimated by measuring the length of 15–30 consecutive clearly visible cells for four different melt pools. Densities were estimated from a 3.45 mm^2^ large image towards the center of the samples with an estimated error of ~ 1% porosity. Area of cracks was calculated by image analysis of four 1.5 mm^2^ areas.

Heat treatment was performed in a VBF-1200X furnace. The samples were sealed under vacuum in quartz tubes after being connected to a Varian SD-91 vacuum pump for 15 min. The samples were quickly put into the pre-heated furnace at 560 °C with less than 5 °C temperature drop and then after 5 min heat treatment quenched in water. Magnetic properties of the samples were analyzed using a Lakeshore 8600 series vibrating-sample magnetometer (VSM), an approximated error of 5% in the measured magnetization as a consequence of uncertainty of the centering of the sample can be assumed. The samples were cut into smaller (~ 0.1 g) cubes and glued in gelatin capsules to avoid the risk of them falling off during the measurement. The equipment could only produce a magnetic field of 1.8 T which was not enough for the sample to reach full saturation.

## Results and discussion

### Coating of the MnAl(C) powder

**Figure 1:**
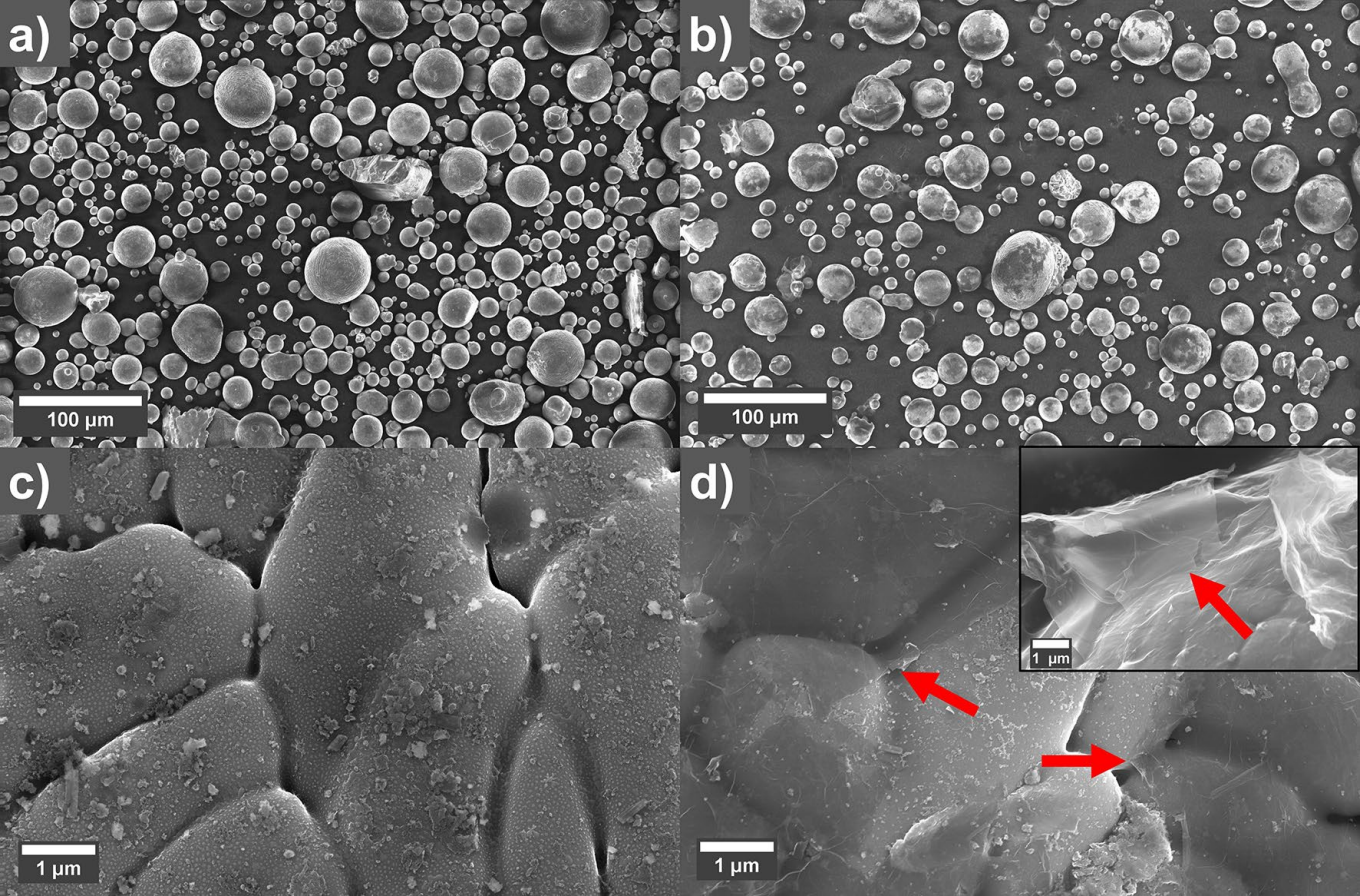
SEM micrographs of MnAl(C) (**a**,**c**) reference and (**b**,**d**) 0.2 wt.% graphene oxide coated powders. The GO-sheets in (**d**) are indicated with red arrows. The inset in (**d**) show a GO sheet that sticks out from the powder particle surface.

After coating the MnAl(C) powder with 0.2 wt.% GO, the powder appeared slightly darker than the un-coated reference (Fig. SI 1c) suggesting a lower reflectance of the GO-coated surface. Scanning electron micrographs (SEM) of MnAl(C) powders before and after coating with graphene oxide (GO) are shown in Fig. 1. The powder particles consist of a large number of grains. There is no observable change to the morphology of the powders after coating, but darker areas, corresponding to GO sheets, can be seen on most of the coated powders (Fig. 1b). Higher magnification of the GO sheets is shown in Fig. 1d. The presence of GO was also shown using Raman spectroscopy of the powders (see Fig. SI 3). There was no observable difference in flowability for the coated and uncoated reference powders, and both had sufficiently good flowability for the powder-bed process. X-ray diffractograms of the powders are shown in Fig. SI 4 in supplementary information.

### Printing process and microstructure of as-printed parts

**Figure 2:**
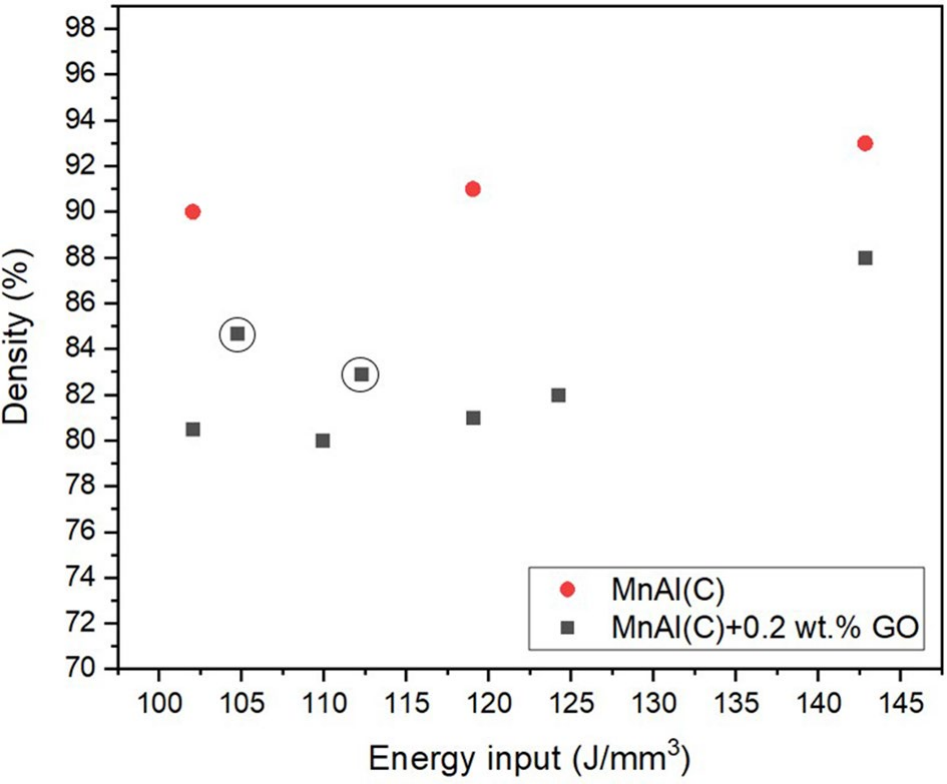
Processing parameter plot comparing energy input vs density of printed parts. Red circles are reference MnAl(C) while black squares correspond to MnAl(C) coated with 0.2 wt.% GO. The circled squares were printed with 22 W laser power while all other samples used 20 W and 200–300 mm/s scanning speed. Hatch distance and layer thickness are 0.07 mm and 0.02 mm, respectively for all samples.

The printing process required rather low laser powers since too high power (50–100 W) lead to a lot of spatter, and possibly in combination with denudation, which resulted in the sample height not keeping up with incremental layer addition of the print process. The samples which were double melted using medium–high laser power were over-melted and areas of the sample, most of the time the edges, would stick up and interfere with the re-coater blade (Fig. SI 5). For the samples which could be completed, the effect of the volume energy input (laser power divided by hatch spacing, layer thickness and scanning speed) compared to the density of the printed parts are shown in Fig. 2. The uncoated reference parts show a higher density for all tested processing parameters compared to coated samples, and densities increase with increasing energy input. Furthermore, the coated powders that were printed with 22 W, but lower scanning speeds (circled black squares in Fig. 2) showed a higher density compared to samples printed with 20 W laser power but similar energy input, indicating that laser power is important. The lower densities for the parts printed with GO coated powders could possibly be explained by the increased spattering that was observed during printing for these powders. The increased spattering from the GO coating that was observed is in agreement with another study using the same type of coating^23^. A potential explanation for the lower density could be that the GO-coating only emphasizes the issues MnAl(C) already have with spattering. More processing parameters would have to be investigated to mitigate this spattering further.

**Figure 3:**
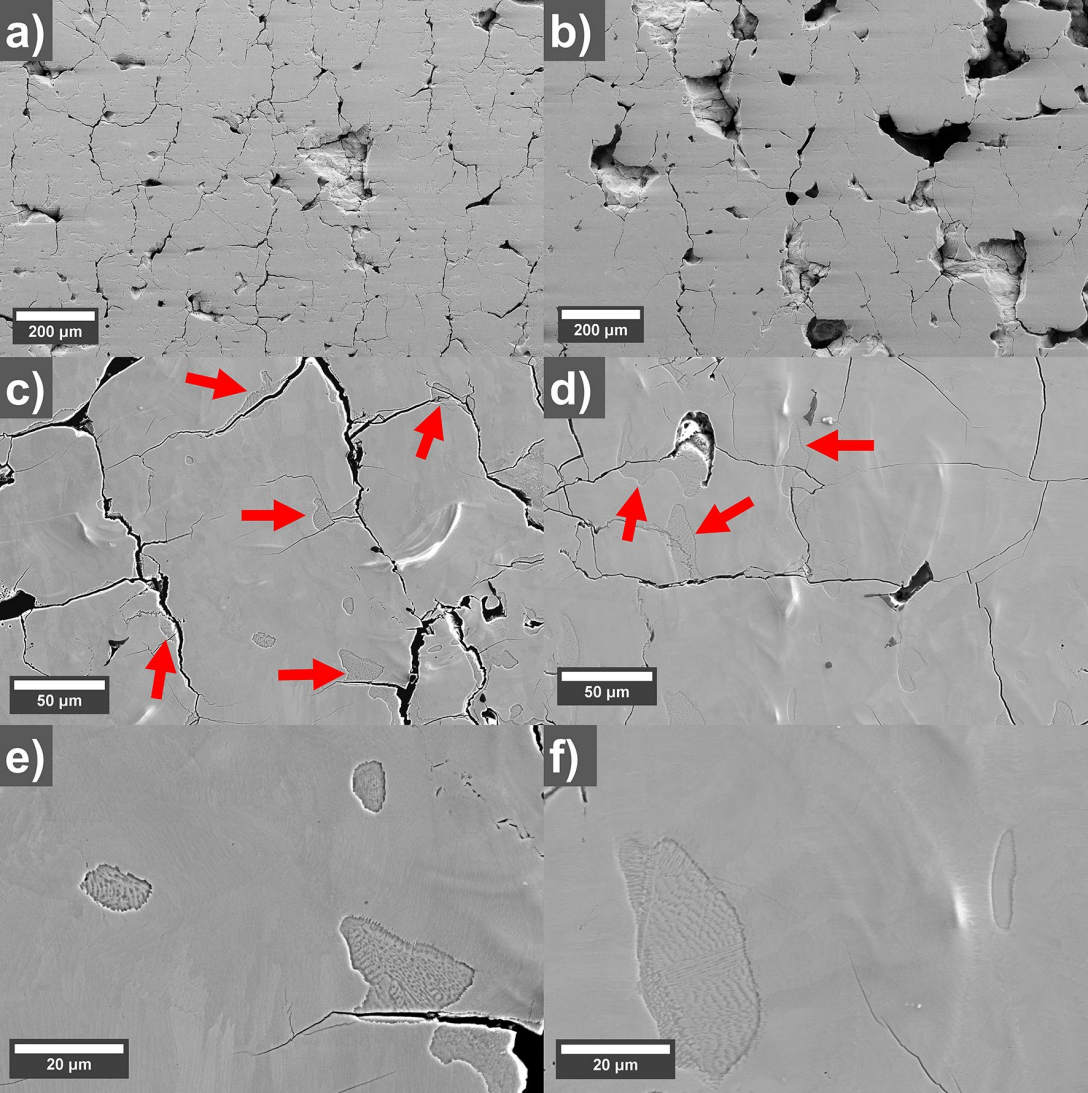
SEM micrographs of polished cross-section of as-printed (**a**,**c**,**e**) uncoated reference (left) and (**b**,**d**,**f**) 0.2 wt.% GO coated samples (right). (**a**,**b**) Low magnification micrographs showing difference in pore morphology and amount of cracks. (**c**,**d**) Show two-phase regions (indicated with red arrows) near cracks and (**e**,**f**) show detailed features of the two-phase regions.

SEM micrographs showing polished cross-sections of printed parts are shown in Fig. 3a,b. The reference sample has a higher density of 91% and a lot of cracks while the sample with 0.2 wt.% GO shows less cracks but more areas of larger pores and a density of 82%. A reduction of cracks could be observed for the sample printed with GO coated powder. The area of the cracks per solid surface (removing larger pores) was estimated to 3.83 (0.31) % for the reference and 2.48 (0.22) % for the 0.2 wt.% GO sample. A likely reason for the reduced cracking could be a higher absorption of the laser light with GO. This is supported by the darker surface color of the GO-coated powder, which should give a higher energy input at a given laser power. A significant difference in energy absorption has been observed previously for GO-coated Cu powder^23^. A previous study on L-PBF of MnAl has shown that a higher laser power indeed reduces cracking^21^. As discussed above, an increase in laser power with uncoated powder only gives rise to either over-melting or further spattering which inhibits the print process from being completed. But the addition of GO allows the process to be stable at the same time as it provides an increase in energy absorption which has positive effects on the crack formation in agreement with Ref.^21^. Hence, the addition of GO has a positive effect on the printing behavior. However, as the densities are lower for the GO coated samples, additional test would need to be done using different concentrations of GO and different scanning strategies, for example using different scanning parameters for the first the second re-melt to optimize the densities. Another potential reason for the reduced crack formation could be that the GO sheets remain in the printed material and inhibits crack propagation, however, no GO sheets could be detected using Raman spectroscopy of the pores in the polished cross-section.

Higher magnification SEM micrographs of the polished cross-sections of as-printed samples (Fig. 3c,d) show features (indicated with red arrows). It seems that cracks either originate or propagate through the larger of these features. There are however some small features that are not connected to pores or cracks. From Fig. 3e,f it can be observed that these features appears to be two-phase region where one of the phases has been etched by the basic polishing suspension of colloidal silica. An explanation for the presence of these can be found when look at the Mn–Al phase diagram^31^. We have also added a calculated Mn–Al phase diagram in supplementary information (Fig. SI 6) using the Thermo-calc software that is zoomed in on this region and the main features are in agreement with Ref.^31^. The ε-phase (Mn_0.55_Al_0.45_ ht) and high-temperature γ-phase (Mn_0.45_Al_0.55_ ht) have a two-phase region, which would also be one of the last to solidify. The basic polishing suspension etches some of the γ-phase in this two-phase region leaving only the ε-phase from the two-phase regions in the matrix of pure ε-phase around it. The Kikuchi patterns when doing electron back-scatter diffraction (EBSD) of these two-phase regions were of poor quality and could not be indexed properly. Areas like this with unindexed Kikuchi patterns could also be observed in the ε-phase in the work of Fang et al.^32^. There seem to be more of these features in the uncoated reference compared to the 0.2 wt.% GO sample, and could be an explanation for the increased cracking in the reference. The presence of different phases in this region would induce stresses during thermal cycling when subsequent adjacent layers are melted which results in crack formation or propagation. The difference of these features between the as-printed uncoated reference and 0.2 wt.% GO samples could be explained by increased heat absorption of the laser that the GO provides during L-PBF processing which would lead to overall higher temperatures and slower cooling which seem to influence the sizes and amount of these features.

**Figure 4:**
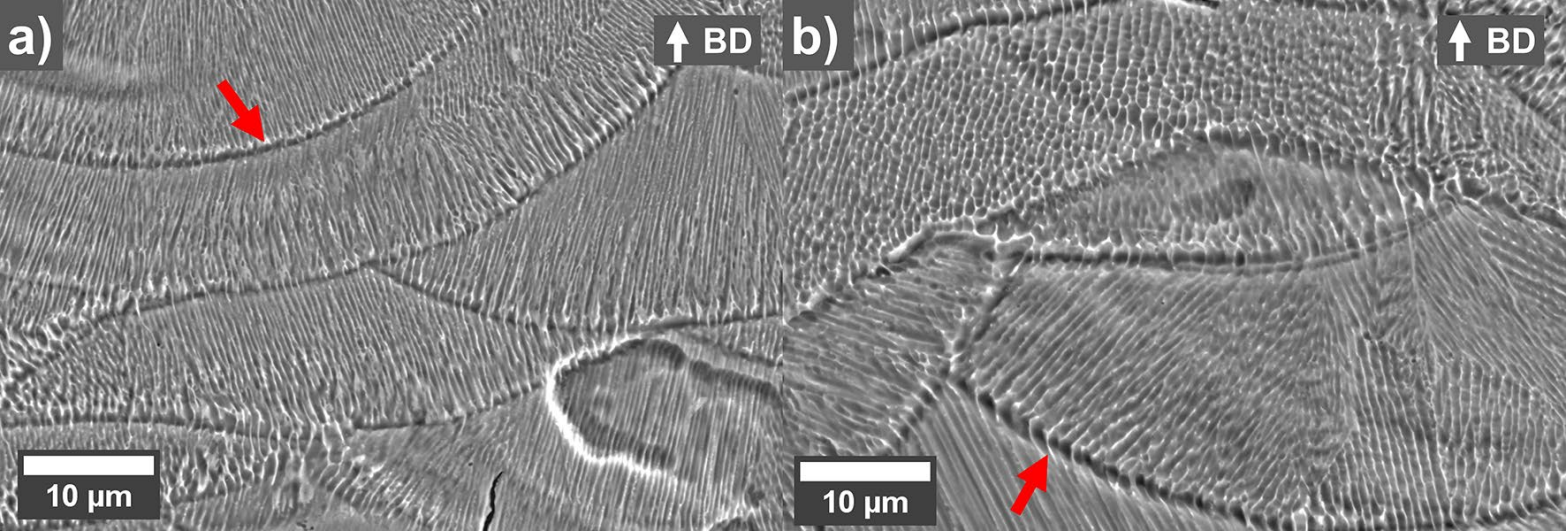
SEM micrographs of etched cross-sections for as-printed (**a**) uncoated reference and (**b**) 0.2 wt.% GO coated samples. Melt pool boundaries (indicated with red arrows) and cellular microstructure can be observed. Build direction (BD) is up in the figure.

The etched cross-sections (Fig. 4) of the as-printed uncoated reference and 0.2 wt.% GO sample show a cellular structure, which can appear to be elongated or more rounded depending on which way the cell has been cut. The walls of the cells have not been etched as much and appear bright in the SEM micrograph. The width of the cells for the uncoated reference was 0.434 ± 0.07 µm while the coated sample showed a much larger cell width of 0.708 ± 0.12 µm. Additionally, the cells seem to grow from the melt-pool boundary towards the middle of the melt pool. However, the coated sample also show more cells that are more parallel to the scanning direction (less elongated cells), which could be due to the nature of the rotating scanning direction or different solidification pathways due to GO affecting either the viscosity of the melt or the energy absorbed by the laser.

**Figure 5:**
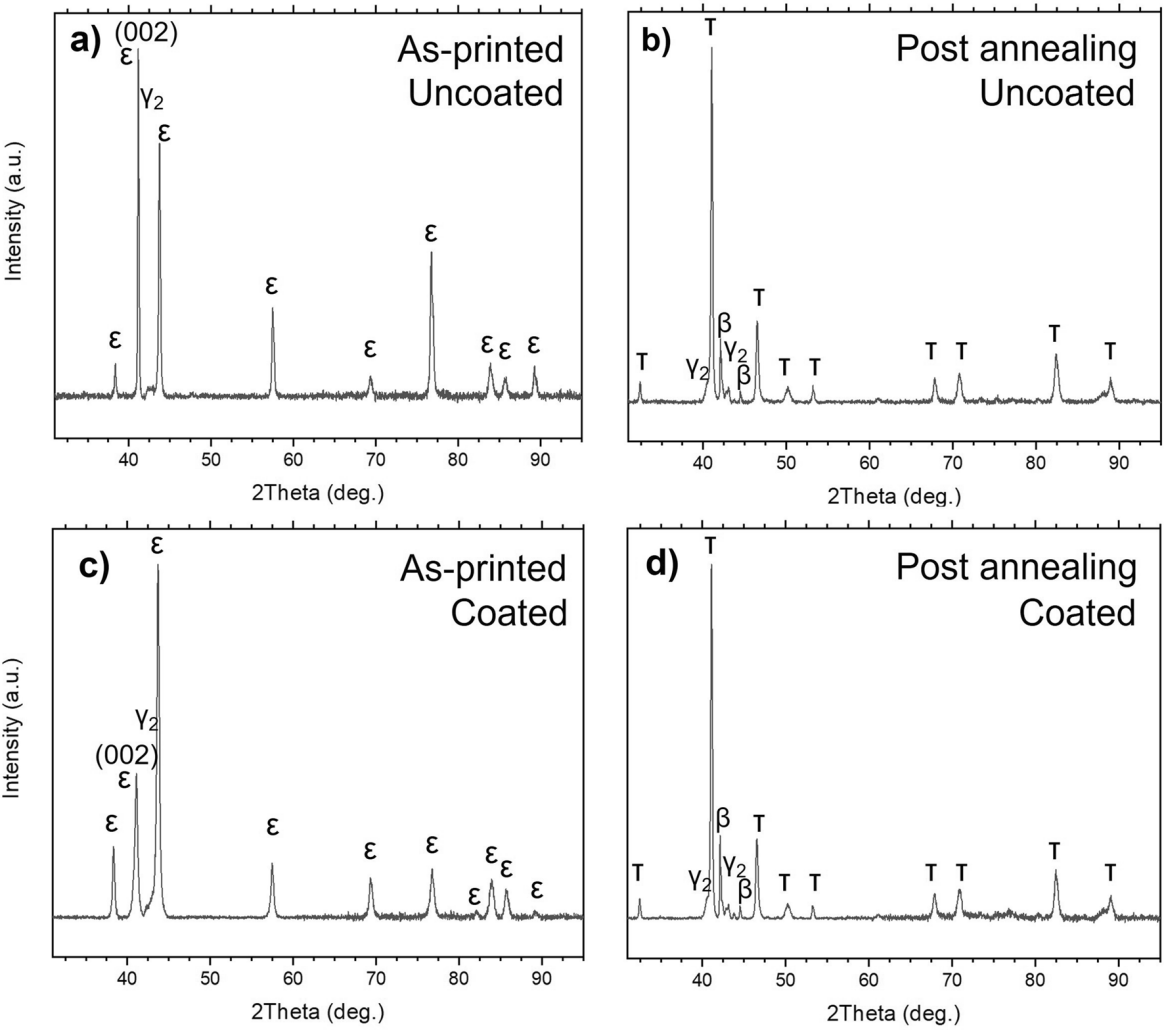
X-ray diffractograms using Cu-Kα radiation of printed samples using 20 W laser power, 240 mm/s scanning speed and a double melting strategy. Reference MnAl(C) of (**a**) as-printed and (**b**) after heat treatment at 560 °C for 5 min. MnAl(C) samples coated with 0.2 wt.% GO (**c**) as-printed and (**d**) after heat treatment at 560 °C for 5 min.

X-ray diffractograms of the as-printed parts using uncoated reference and the GO-coated powder are shown in Fig. 5a,c. Both samples show the hexagonal ε-phase as a majority phase and small amounts of the γ_2_-phase. Refined phase fractions are shown in Table 1, which show that the amount of the γ_2_ phase is similar at around 8.5 wt.% for both samples. There is a stronger $\langle {001} \rangle$ texture along the build direction for the uncoated reference in the as-printed parts which is consistent with previous reports^22^. This was however not observed for the coated samples.

**Table 1:** Calculated phase fractions (wt.%) for the as-printed and annealed uncoated reference and coated sample. Approximately 1 wt.% error for all phase fractions.

Sample	Phase fractions (wt.%)			
	ε	γ_2_	τ	β
As-printed uncoated ref	92	8	–	–
As-printed coated	91	9	–	–
Annealed uncoated ref	1	14	77	8
Annealed coated	4	14	73	9

### Annealing of printed MnAl(C)

**Figure 6:**
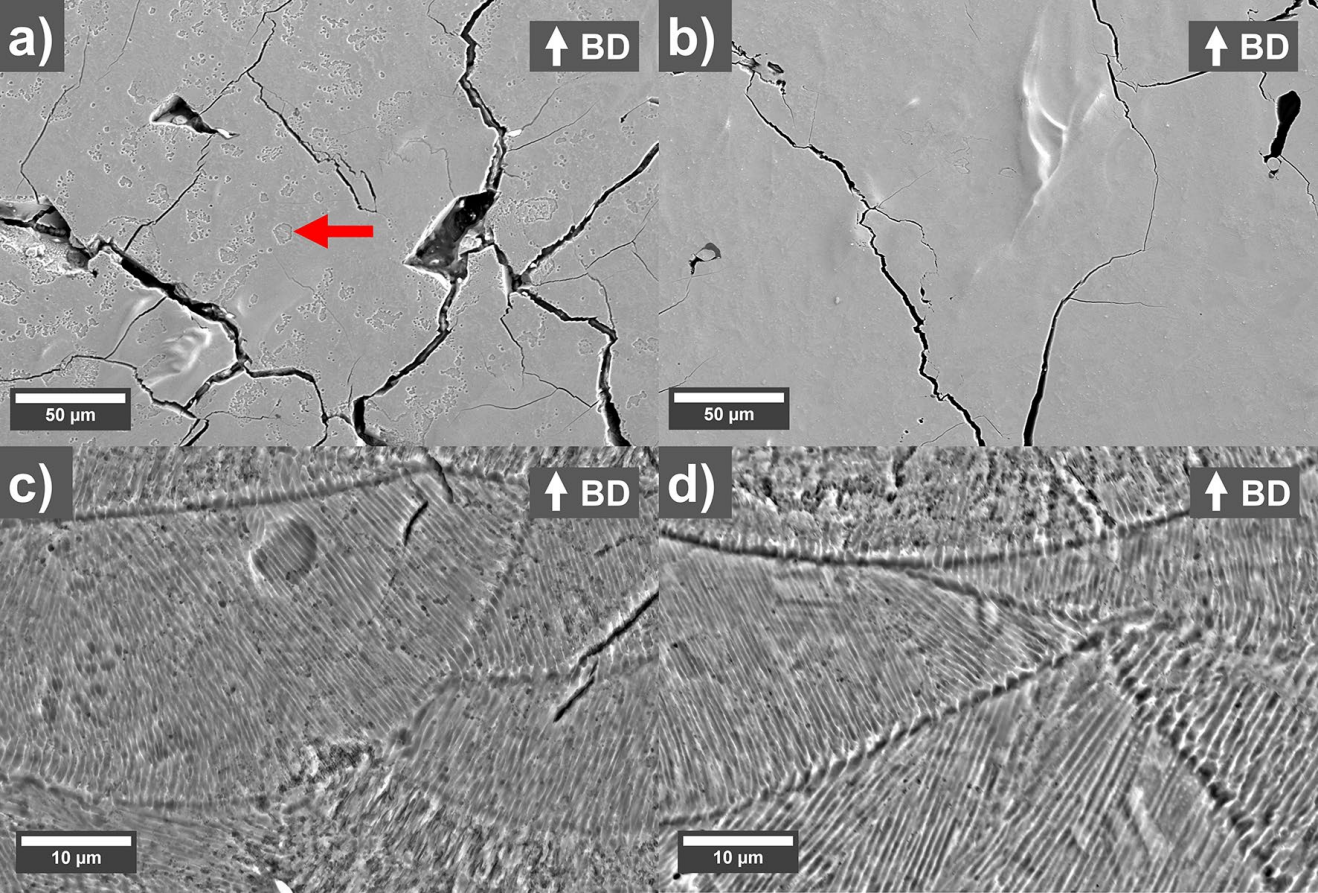
SEM micrographs of cross-sections of annealed (**a**,**c**) uncoated reference (left) and (**b**,**d**) 0.2 wt.% GO (right) parts heat-treated at 560 °C for 5 min. (**a**,**b**) Show unetched cross-sections while (**c**,**d**) show etched cross-sections. Build direction (BD) is up in the figure.

The printed samples were annealed at 560 °C for 5 min to form the ferromagnetic metastable τ-phase X-ray diffractograms in Fig. 5b,d shows that after annealing, the τ-phase is present in both samples together with two minority phases consisting of β-phase and γ_2_-phase and some trace amounts of ε-phase suggested by the shoulder on the left size of the largest peak from the τ-phase, suggesting a near complete transformation of the ε-phase after the annealing step. The refined phase fractions of the annealed samples are shown in Table 1. The texture difference in the in the ε-phase of the as-printed samples were not retained in the τ-phase after annealing, which is consistent with previous reports^22^. Figure 6 shows high magnification SEM of annealed uncoated reference sample and GO-coated sample. As can be seen, The cross-sections of the polished heat-treated samples (Fig. 6a,b) look different to the as-printed (Fig. 3). For the 0.2 wt.% GO sample very few of these features could be observed, but for the uncoated reference many features could be observed, these looked much more even than for the as-printed which would suggest that this is not a two-phase region but a pure γ_2_-phase. According to the XRD refinement in Table 1, the samples should contain similar amount of γ_2_-phase, and they were polished at the same time. The difference could possibly be caused by the finer cellular structure of the uncoated reference sample leading to different rates of grain growth of the new phases during heat treatment. The cellular structure observed in the as-printed samples remains in some form after annealing (Fig. 6c,d) and the cells are of similar widths compared to before annealing for both the uncoated reference and the coated sample. Additionally, melt-pool boundaries could still be observed in both samples. This means that 5 min annealing time is not enough to remove all microstructural features remaining from the printing process.

### Magnetic properties of annealed MnAl(C) samples

**Figure 7:**
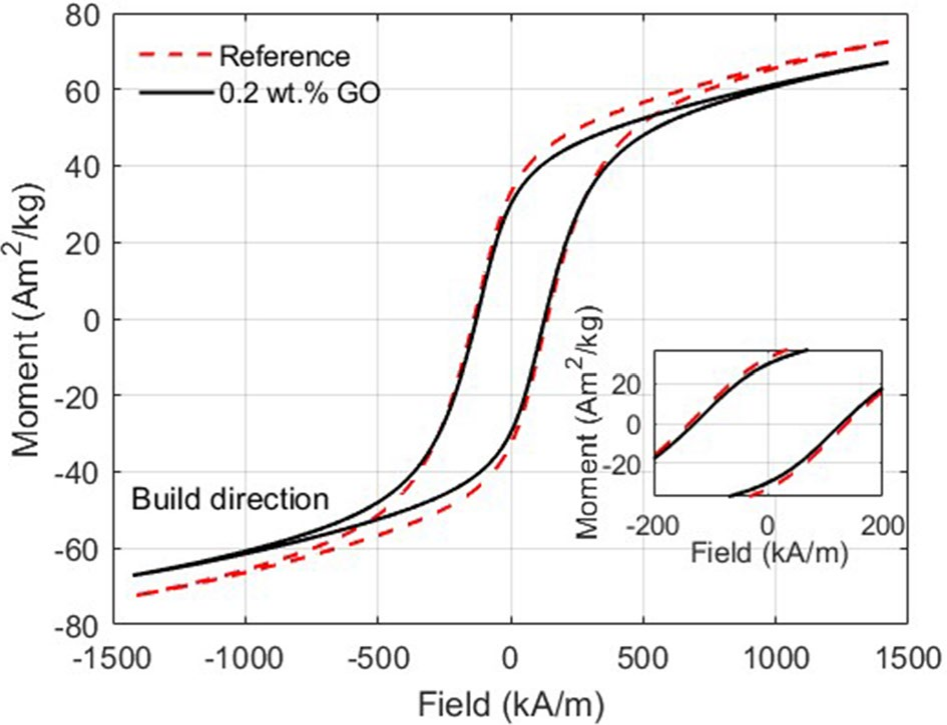
Hysteresis loops measured with VSM of printed MnAl(C) samples showing reference (red dashes) and 0.2 wt.% GO (full black line) after heat treatment at 560 °C for 5 min, measured with the field along an edge of the cube in the build direction.

The magnetic properties of the printed heat-treated reference and 0.2 wt.% GO sample can be seen in the hysteresis loops in Fig. 7. Compared to their respective powder feedstock (Fig SI 7), the annealed printed samples have a high magnetic saturation and coercivity. The reference showed a coercivity of 139 kA/m and a remanence of 33 Am^2^/kg while the 0.2 wt.% GO sample showed a slightly lower coercivity of 130 kA/m and a remanence of 30 Am^2^/kg. This is an increase in remanence for both samples compared to previous reports of L-PBF of MnAl(C)^22^, but a decrease in coercivity. However, a direct comparison between results is discouraged in this case since the exact geometry of the sample measured is not reported, and the data in the present study is presented without correction for demagnetizing effects. The magnetic properties of the printed samples were isotropic when comparing the measurements parallel (Fig. 7) and perpendicular (Fig. SI 8) to the building direction. The slightly lower coercivity and remanence for the coated sample correlates well with the lower phase fraction of the τ-phase and could possibly be improved by a different heat treatment. The different behavior during the heat treatment could be explained by the smaller cell structure of the uncoated reference. Further research is however needed to establish a more detailed description of the influence of microstructure on the magnetic properties and how to optimize them.

## Conclusions

The MnAl(C) powder was coated with graphene oxide (GO) using attractive electrostatic forces between the positively charged native metal oxide surface and the negatively charged GO sheets in a wet chemical process at pH 7.4. The L-PBF printed parts using powders coated with 0.2 wt.% GO showed significantly reduced cracking. However, the densities of the printed parts using the coated powder decreased from 91 to 82%. Both the uncoated reference powder and the coated powder yielded the ε-phase in the as printed parts and small amounts of the γ_2_-phase. Two-phase regions of a mix of ε- and γ_2_-phase in the surrounding pure ε-phase could be observed in the as-printed parts that seemed to be more prominent in the uncoated reference samples and could also be linked to cracks. The addition of GO also suppressed the $\langle {001} \rangle$ fibre texture along the build direction for the ε-phase. After 560 °C for 5 min the ferromagnetic τ-phase was obtained for both samples in addition to small amounts of the β- and the γ_2_-phase. The reference and the 0.2 wt.% GO samples and showed similar magnetic properties. The reference had a remanence of 33 Am^2^/kg and a coercivity of 139 kA/m while the 0.2 wt.% GO sample showed a remanence of 30 Am^2^/kg and coercivity of 130 kA/m, which could be explained by a slightly lower τ-phase content. This study shows that GO can be used as an effective method of reducing cracks in L-PBF of MnAl(C) without affecting the magnetic properties. Further studies are needed to investigate how other printing parameters, optimized for the coated powders, can be used to reduce spattering and increase the density, possibly investigating double-melting but with different parameters for the first and second scan.

### Supplementary Information

Supplementary Information.

## Data Availability

The datasets used and/or analyzed during the current study are available from the corresponding author on reasonable request.
