# Supplementary material for: Crack reduction in laser powder bed fusion of MnAl(C) using graphene oxide coated powders
Source: Sci Rep. 2024 Jan 11;14:1142. doi: 10.1038/s41598-024-51283-5 (PMC10784453; doi:10.1038/s41598-024-51283-5)
Supplement: Supplementary file 1 — Supplementary Information. [file 41598_2024_51283_MOESM1_ESM.docx]

**Supplementary information**

**Table SI 1.** Particle size distribution of as-atomized MnAl(C) powder determined from sieve analysis. Only the powder fractions below 63 µm was used for L-PBF printing.


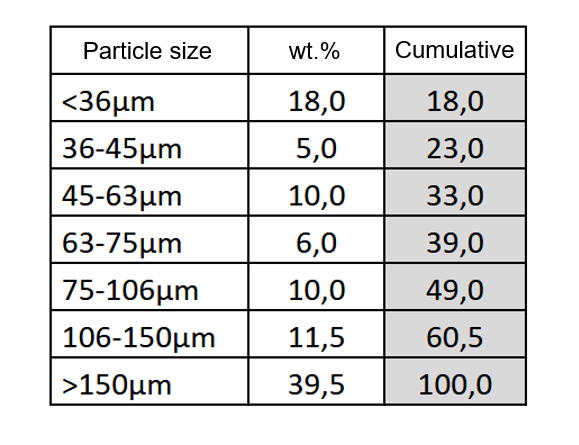


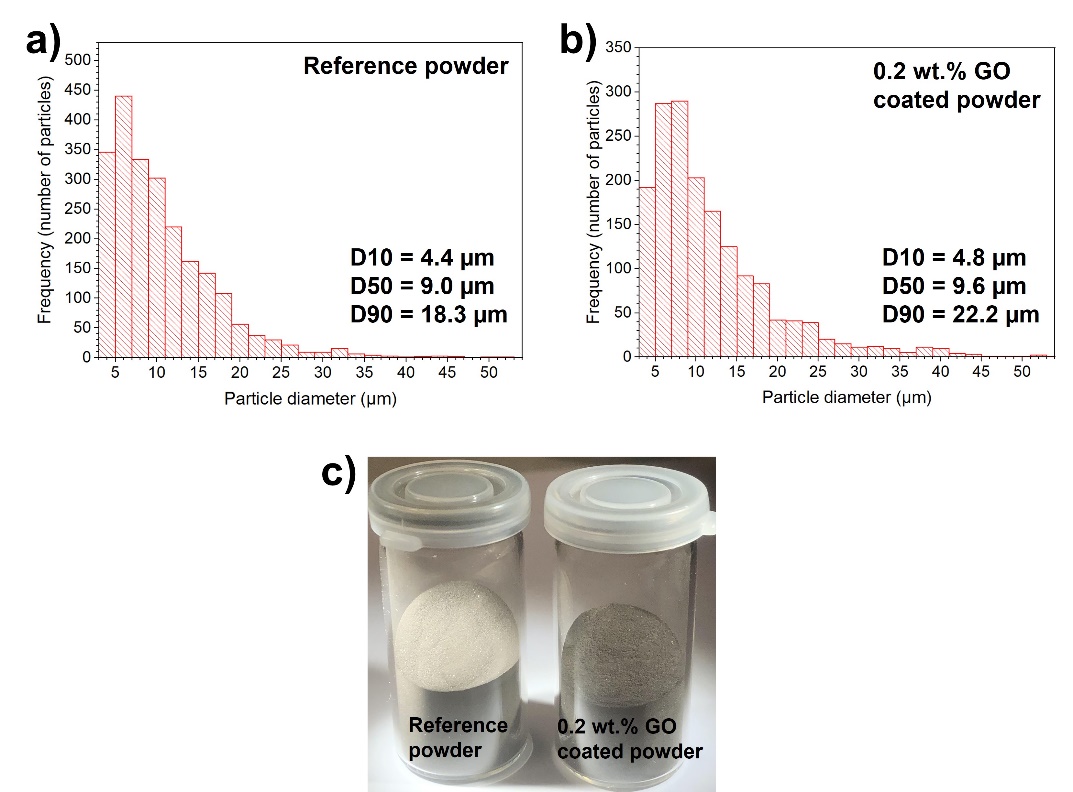


**Figure SI 1.** Particle-size distribution (PSD) for powders used for printing (<63 µm) showing a) reference powder and b) 0.2 wt.% graphene oxide (GO) coated powder with number weighted D10, D50 and D90 values. c) show the color difference for reference (left) and coated (right) powder. PSD was calculated from image analysis in ImageJ of SEM images of powder particles.

**
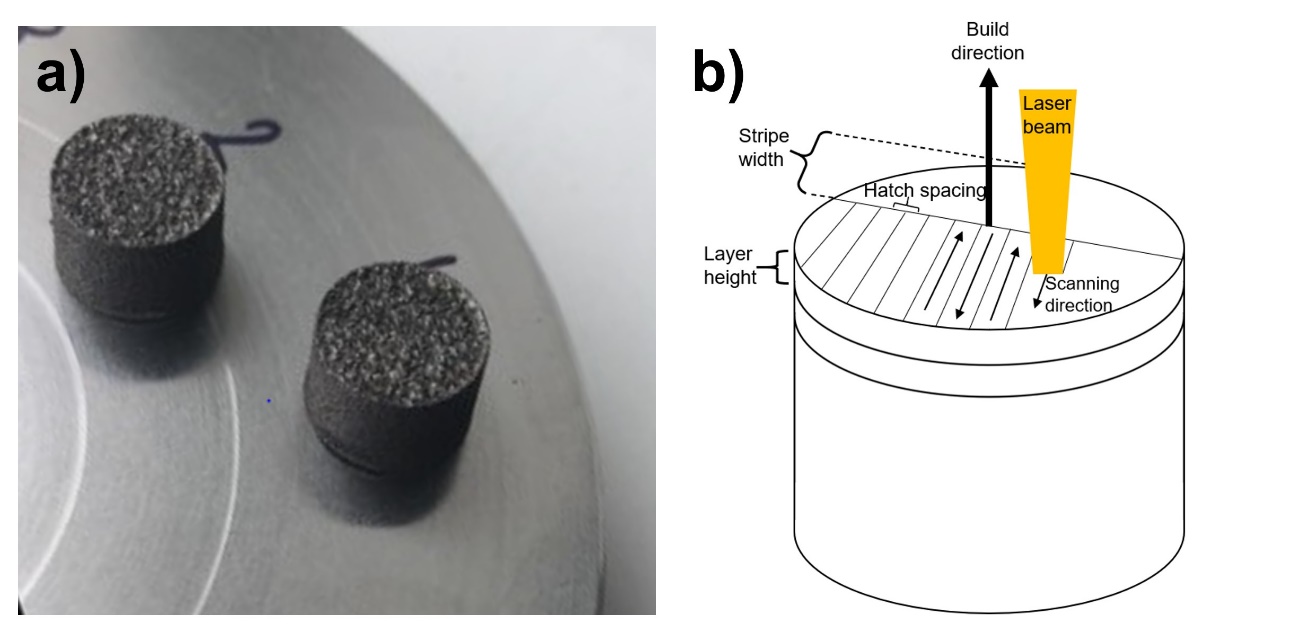
**

**Figure SI 2.** a) Image of samples using graphene oxide coated powders printed with 20 W laser power and 240 mm/s scanning speed using double melting strategy, b) show a schematic describing the different printing parameters. Sample dimensions is 8 mm diameter and 6 mm height.


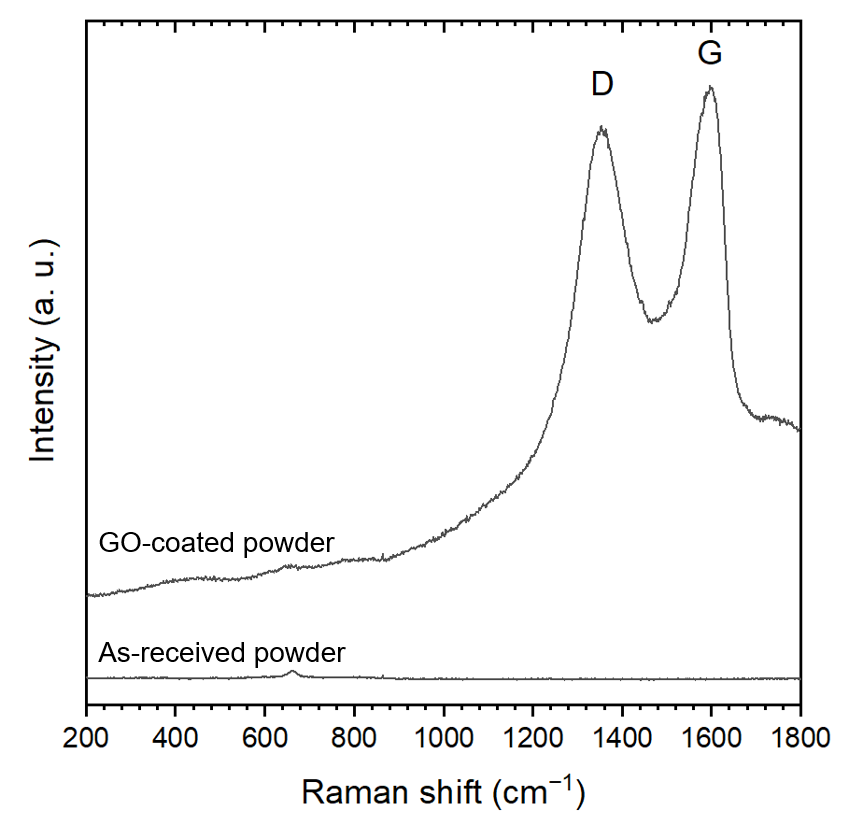


**Figure SI 3.** Raman spectrum of the surface of the graphene oxide (GO) coated MnAl(C) powder showing the characteristic D- and G-band for GO.


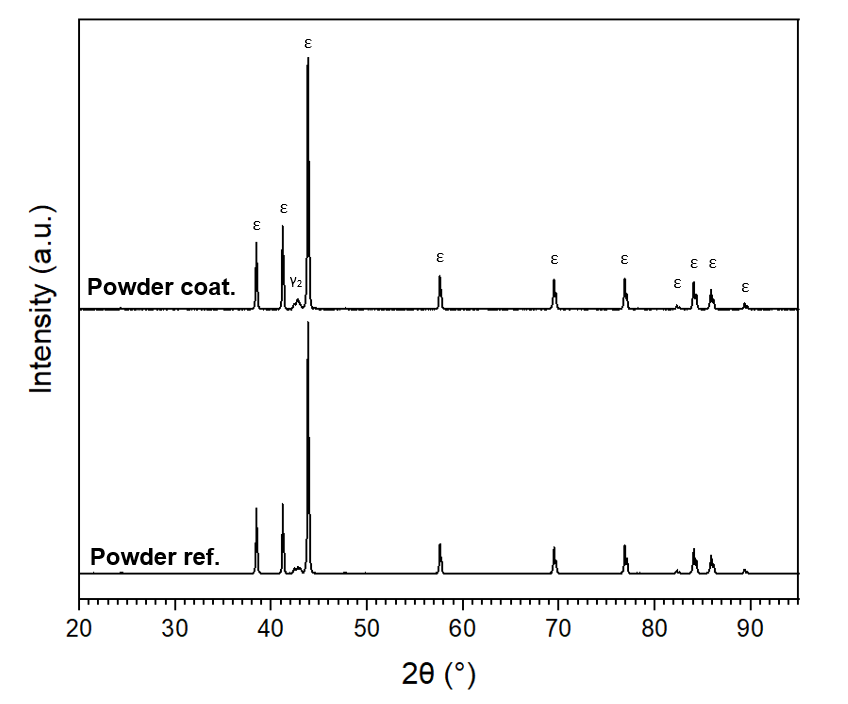


**Figure SI 4.**  X-ray diffractograms of reference powder (ref.) and 0.2 wt.% graphene oxide coated powders (coat.). The powders consist mostly of the ε-phase but also small amounts of the γ_2_ phase.


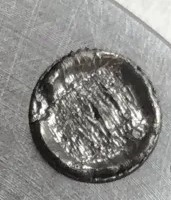


**Figure SI 5.** Image of a sample using 50 W laser power and 300 mm/s scanning speed that were double melted. It has elevated edges which interfered with the re-coated blade which detached a piece from the sample.


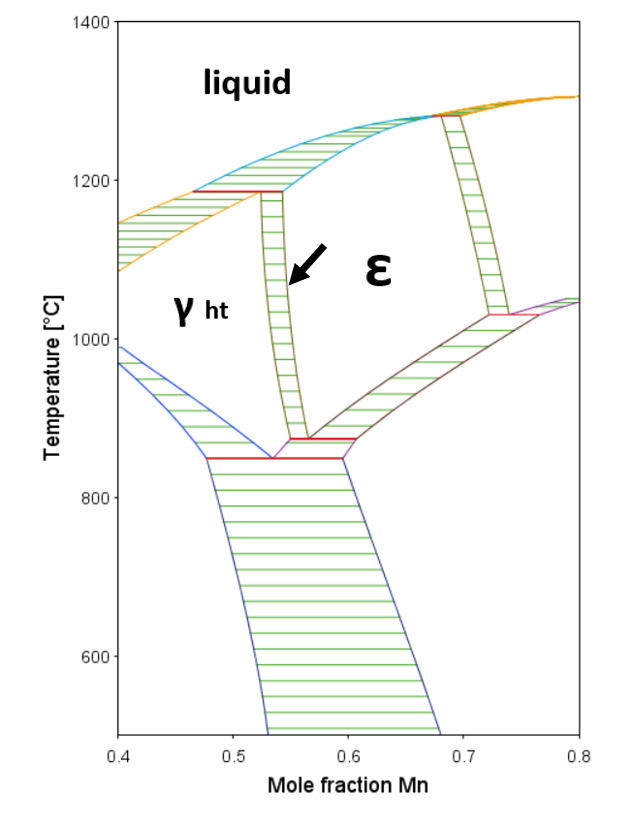


**Figure SI 6.** Binary phase diagram of the Mn-Al system simulated in Thermo-calc software. Two-phase region between the ε-phase and the high temperature γ-phase is indicated with an arrow.

**
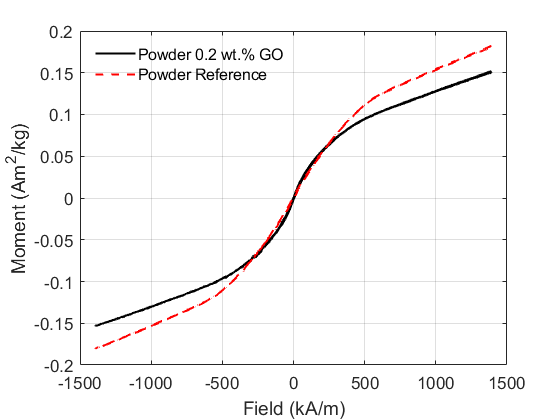
**

**Figure SI 7.** Hysteresis loops of the two feedstock powders measured with VSM.


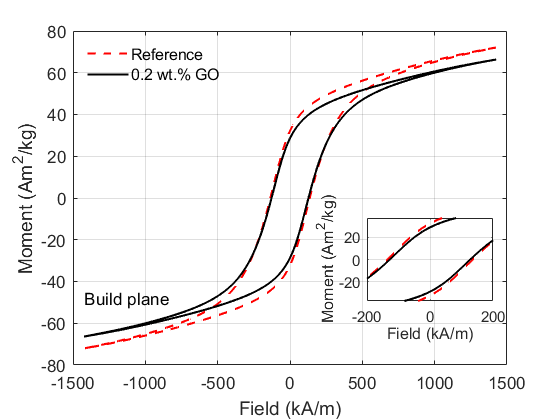


**Figure SI 8.** Hysteresis loops of printed MnAl(C) samples measured with the field along an edge of the cube perpendicular to the build direction measured with VSM.
